# Deciphering GABBR1-centered drug targets to fight viral infection with preexisting diabetes: Targeting GABBR1 for viral infection and diabetes

**DOI:** 10.3724/abbs.2023249

**Published:** 2023-11-15

**Authors:** Xiangqi Li, Xuemei Zhao, Li Peng, Hong Du, Shiwei Chen, Xia Chen

**Affiliations:** 1 Department of Endocrinology and Metabolism Gongli Hospital Naval Medical University Shanghai 200135 China; 2 Department of General Practice Hudong Community Health Service Centre Shanghai 200129 China; 3 Department of Intervention Gongli Hospital Naval Medical University Shanghai 200135 China

Viral infections are a widespread ocial problem. The biggest impact right now is asymptom-triggering COVID-19 [1]. It is also termed “WARS” because it resembles a challenge of wars that mankind is facing [2]. COVID-19 has the potential to develop into severe illnesses such as systemic inflammatory response syndrome and sepsis [3]. Severe cases or deaths of COVID-19 are characterized by advanced age, male sex, and having underlying diseases, including cardiovascular disease, cancer, obesity, and type 1 or 2 diabetes mellitus [4]. Underlying diabetes mellitus is common among patients with COVID-19 admitted to ICUs [5]. Even after 3 years, there are no specific drugs available to treat COVID-19. This is even worse for patients with COVID-19 with diabetes. Therefore, determining the targets that can be used to develop drugs to simultaneously treat both diabetes and COVID-19 is a particularly valuable strategy for saving lives.

We have previously shown that bitter medicines targeting the bitter taste receptor TAS2R may restore the imbalance of inflammation/immunity and enhance the host’s ability to fight COVID-19 [ 2, 6]. GABBR1, a predicted interacting protein of TAS2R14, encodes a receptor for gamma-aminobutyric acid (GABA), which is the main inhibitory neurotransmitter in the mammalian central nervous system and is commonly used as an antianxiety drug. We suspected that it could also be closely related to inflammation/immunity and could be used as a target against COVID-19. In fact, a growing body of evidence demonstrates its immunoregulatory activities [7].

In this study, we evaluated GABBR1 as a shared target between diabetes and viral infection (focusing on COVID-19) for drug development on the basis of numerous brilliant databases and bioinformatics tools (for details, see Supplementary Methods). We enlisted the tool DISEASES to parse GABBR1–disease associations mined from the literature. A total of 155 types of disease associations were uncovered ( Supplementary Data 1), and diabetes and viral infections were included ( Supplementary Figure S1A). These results supported our original idea. Then, we designed a protocol for analyzing GABBR1-related biological functions, molecular mechanisms, and drug actions ( Supplementary Figure S1B).

We first parsed the biological roles required for the involvement of GABBR1 in coexisting diseases using a co-RNA dataset. A total of 113 co-RNA genes of GABBR1 were extracted in the liver from the online GEPIA database. The DAVID tool was used to dissect the functional associations of GABBR1 by analyzing coRNAs ( Supplementary Data 2). GAD-related terms exhibited GABBR1 functional associations with diabetes ( Supplementary Figure S2A). GO-BP analysis indicated that GABBR1 can be involved in several biological processes ( Supplementary Figure S2B). The term GO-CC implied that the functions of GABBR1 were related to wide cellular regions ( Supplementary Figure S2C). GO-MF reflects several molecular functions of GABBR1 ( Supplementary Figure S2D). From KEGG, we determined the relationships of GABBR1 with viral infections, such as “HTLV-I infection” ( Supplementary Figure S2E). Obviously, the results of HIV-INTERACTION analysis further support its involvement in viral infections ( Supplementary Figure S2F).

Then, we dissected the involvement of GABBR1 in diabetes and viral infections using a ceRNA dataset. Because ceRNA can reflect direct molecular interactions, we extracted 500 ceRNA genes of GABBR1 from the ENCORI database to parse the functions of GABBR1 to confirm its potential roles in diabetes and viral infections ( Supplementary Data 3). The top 20 terms of GAD showed that GABBR1 can participate in various diseases, including “type 2 diabetes” ( Supplementary Figure S3A). KEGG annotations revealed its role in viral infections, such as “HTLV-I infection” ( Supplementary Figure S3B). Certainly, the existence of HIV-INTERACTION terms further supports its involvement in viral infections ( Supplementary Figure S3C). In total, we found four terms related to diabetes and four comments on viral infections ( Supplementary Figure S3D). These data revealed the functional notes of ceRNAs by GAD, KEGG, and HIV-related analysis, supporting the roles of GABBR1 in diabetes and viral infections.

Next, we constructed a ceRNA-associated molecular network involved in diabetes and viral infections for GABBR1. Based on ceRNA analysis, to identify shared genes between diabetes and viral infections, 84 diabetes-related genes and 49 virus-related genes were intersected. We obtained 15 intersecting genes ( Supplementary Figure S4A). A total of 113 co-RNA genes were enlisted to intersect these 15 genes, and we identified two cross genes, *PDGFRB* and *WNT2B* ( Supplementary Figure S4B). A total of 63 miRNAs of PDGFRB and 52 miRNAs of WNT2B had the ability to form ceRNAs with GABBR1 ( Supplementary Data 4 and Supplementary Figure S4C). These two coding RNAs had 21 shared miRNAs ( Supplementary Figure S4C,D). The expression profiling of these 21 miRNAs showed that only one miRNA, hsa-miR-19b-3p, shared high expression and another miRNA, hsa-miR-19a-3p, shared medium expression between NGS and ARRAY ( Supplementary Figure S4E). To provide further evidence for these network molecules, we queried them in InnateDB. We found that all are registered as innate immune genes, which supports their ability to participate in viral infections ( Supplementary Figure S4F). Therefore, we concluded that we obtained a shared ceRNA network between diabetes and viral infections, which consisted of GABBR1, PDGFRB, WNT2B, and hsa-miR-19b/a-3p ( Supplementary Figure S4G).

We also revealed the involvement of GABBR1 in diabetes and viral infections by an interRNA dataset. The interRNA indicates a new gene expression regulatory mechanism through direct RNA-RNA interaction. We extracted 100 interRNA genes of GABBR1 from the ENCORI repository to confirm the roles of GABBR1 in diabetes and viral infections ( Supplementary Data 5). Nine terms of GAD revealed that GABBR1 can be involved in several diseases, including diabetes ( Supplementary Figure S5A). Four annotations of KEGG disclosed its role in viral infections, such as “Influenza A” ( Supplementary Figure S5B). Five terms of HIV-INTERACTION further supported its involvement in viral infections ( Supplementary Figure S5C). Generally, we identified one note related to diabetes and six annotations related to viral infections ( Supplementary Figure S5D). On the basis of these results, we determined the functional terms of interRNAs by GAD, KEGG, and HIV-related analysis, which further provided support for the involvement of GABBR1 in diabetes and viral infections.

We further analyzed interRNA-related signaling pathways involved in diabetes and viral infections for GABBR1. According to the above-described interRNA data, 13 genes related to diabetes and 27 genes related to viral infections were enlisted to intersect shared genes between diabetes and viral infections. We obtained six overlapping genes, *GSK3B*, *STAT1*, *HMGA1* , *SMARCA4*, *AKT2*, and *NAMPT* ( Supplementary Figure S6A). A total of 1000 co-RNA genes were recruited to cross the above-obtained six genes, and we obtained two hub genes, *HMGA1* and *SMARCA4* ( Supplementary Figure S6B). These two genes had low free energy and high alignment scores, which indicated a strong interaction with GABBR1 ( Supplementary Figure S6C). To provide additional information on their biological roles, we queried them in InnateDB and found them registered as innate immune genes, which supports their roles in viral infections ( Supplementary Figure S6D). On the basis of these results, we achieved a shared interRNA mechanism between diabetes and viral infections, which consisted of GABBR1, HMGA1, and SMARCA4 ( Supplementary Figure S6E).

We then revealed signaling cells of GABBR1-centered network genes in diabetes and viral infections by profiling single-cell expression. We checked the expression level of GABBR1-centered pathway genes in the liver at the single-cell level. GABBR1 was expressed in all types of liver cells, including B cells, cholangiocytes, endothelial cells, erythroid cells, hepatic stellate cells, hepatocytes, Kupffer cells, and T cells. Among them, B cells, endothelial cells, and erythroid cells showed high expression, whereas cholangiocytes, hepatic stellate cells, and Kupffer cells showed medium expression ( Supplementary Figure S7A). PDGFRB showed high expression in hepatic stellate cells and medium expression in cholangiocytes, endothelial cells, hepatocytes, and T cells ( Supplementary Figure S7B). WNT2B showed high expression in endothelial cells, hepatic stellate cells, and hepatocytes and medium expression in B cells, cholangiocytes, Kupffer cells, and T cells ( Supplementary Figure S7C). HMGA1 showed high expression in most cell types, except hepatocytes, with the highest expression in erythroid cells and B cells ( Supplementary Figure S7D). SMARCA4 showed a high expression level in most cell types, except for moderate expression levels in hepatic stellate cells and hepatocytes ( Supplementary Figure S7E). These results indicate that all genes exhibited medium or high expression in cholangiocytes, endothelial cells, hepatic stellate cells, and T cells, where they may signal each other in diabetes and viral infections.

Finally, we refined drugs from chemicals targeting GABBR1-centered signaling molecules for treating diabetes and COVID-19. The CTD can reveal how chemicals affect human health. We used this database to determine which environmental exposures can target our network genes and found 153 such chemicals ( Supplementary Data 6). Because of the excessive number of chemicals for individual investigation, we selected 27 chemicals that can target two or more genes of our network for further analysis ( Figure 1A). Interestingly, we found some drugs in clinical trials that matched our expectations. We present the most advanced clinical trial results from DrugBank. Five drugs, cyclosporine ( Figure 1B), estradiol ( Figure 1C), progesterone ( Figure 1D), nicotine ( Figure 1E), and epigallocatechin gallate ( Figure 1F), can treat both diabetes and viral infections, including COVID-19. Regarding cyclosporine, it has completed its clinical trials at phase IV for “Type 2 diabetes Mellitus Nos New Onset,” “Diabetic Nephropathy/Type 2 Diabetes Mellitus,” “Maintenance of Liver Transplant Patients With New Onset Diabetes,” “HCV Infection,” “Adenoviral Keratoconjunctivitis,” “COVID-19,” “HIV Infection,” and “Polyomavirus Infections,” except “Type 1 Diabetes Mellitus” at phase II and “Respiratory Syncytial Virus” at phase I ( Figure 1B). Four drugs, genistein, tretinoin, valproic acid, and hydrogen peroxide, are used to treat COVID-19 ( Figure 1G). Nevertheless, four drugs, arsenic trioxide, tretinoin, valproic acid, and topotecan, can be used to treat HIV infection ( Figure 1H). Obviously, there are several drugs in clinical trials among 153 chemicals that may treat both diabetes and viral infections. Scientists interested in these drugs may retrieve them from DrugBank.

**Figure 1:**
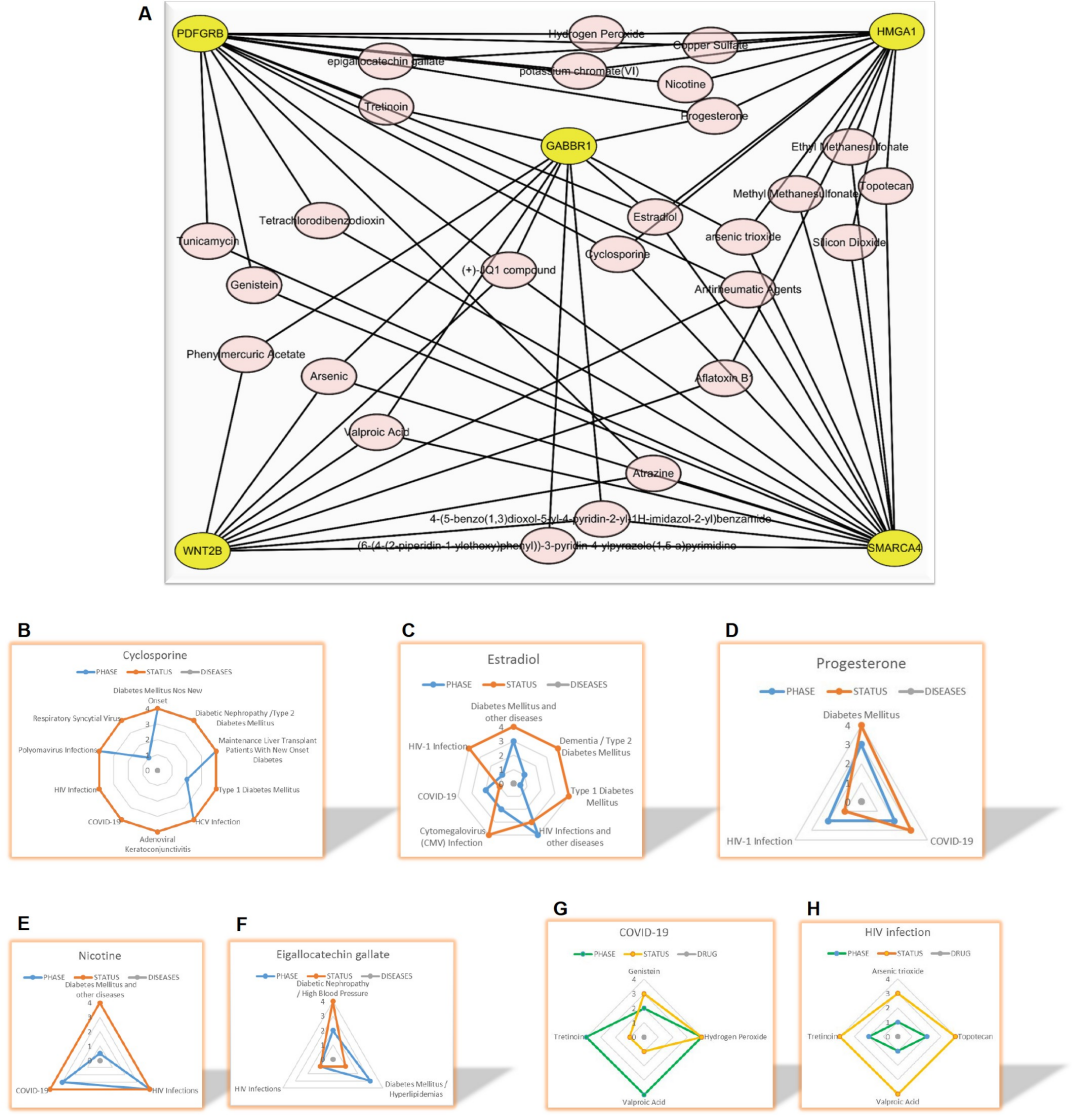
Figure 1 Extraction of drugs from chemicals targeting GABBR1-mediated signaling molecules (A) The intersection chemicals that can target our five network genes. (B) Cyclosporine can clinically treat diabetes and viral infections, including COVID-19. (C) Estradiol can clinically treat diabetes and viral infections, including COVID-19. (D) Progesterone can clinically treat diabetes and viral infections, including COVID-19. (E) Nicotine can clinically treat diabetes and viral infections, including COVID-19. (F) Epigallocatechin gallate can clinically treat diabetes and viral infections, including COVID-19. (G) Genistein, tretinoin, valproic acid, and hydrogen peroxide can clinically treat COVID-19. (H) Arsenic trioxide, tretinoin, valproic acid, and topotecan can clinically treat HIV infection.

Although large numbers of drugs have been screened, a miracle drug targeting COVID-19 is lacking. Emerging evidence indicates various long-lasting complications after discharge from the hospital, which suggests wide-ranging immunoinflammatory derangements in patients with COVID-19 [ 8, 9]. The risk of severe disease or death among these patients is higher in men, older individuals, and those with underlying diseases [ 4 , 10]. Thus, the consequences of COVID-19 depend on the host’s own response. Understanding the pathophysiology underlying the host response to COVID-19 will be critical to developing personalized treatment and management strategies for patients. Moreover, a patient with preexisting diabetes undoubtedly represents a typical case. Here, we found that 153 chemicals can disturb this network. Because of this excess number, we could not dissect them one by one. We describe only the chemicals targeting at least two coding genes of our molecular network. A total of 11 valuable drugs were filtered out from these chemicals ( Figure 1B–H), among which five drugs (cyclosporine, estradiol, progesterone, nicotine, and epigallocatechin gallate) can actualize the potential to treat not only diabetes but also viral infections, including COVID-19 ( Figure 1B–F), which provides strong support for our proposal. Another six drugs are used to treat viral infections ( Figure 1G,H), among which four drugs (genistein, tretinoin, valproic acid, and hydrogen peroxide) are undergoing clinical trials for COVID-19. We can also speculate that these six drugs treating viral infections could also play a role in diabetes, which deserves further investigation. We have previously proposed that host-directed therapy using bitter medicines (HDT-BM) is a beneficial strategy to fight COVID-19 [ 2, 6]. In fact, we extracted five drugs in this study, progesterone, nicotine, epigallocatechin gallate, genistein, and hydrogen peroxide, all of which are bitter. Moreover, all these drugs are under clinical trials for COVID-19 ( Figure 1). This observation not only confirms the effectiveness of our previous HDT-BM but also strongly supports the valuable significance of our identified target molecules in this study. GABA receptors have been reported in the literature as potential targets for COVID-19 therapy, and related agonists may be enlisted to address COVID cases (DOI: 10.1016/j.csbj.2020.11.056; DOI: 10.3390/biomedicines11020254), which supports the results of our paper. Additionally, there are many articles on GABA alleviating both type 1 and type 2 diabetes when we start with a PubMed search for “GABA, T2D” or “GABA, T1D”, which also strongly support our results.

In summary, we evaluated GABBR1 as a shared target between diabetes and viral infections for drug development. Using brilliant databases and bioinformatics tools, we identified GABBR1-centered ceRNAs formed by PDGFRB and WNT2B via hsa-miR-19-3p and RNA-RNA interactions with HMGA1 and SMARCA4 as shared molecules between diabetes and viral infection ( Figure 2). We next identified the signaling cells of cholangiocytes, endothelial cells, hepatic stellate cells, and T cells by profiling their single-cell expression. Then, we used CTD to determine whether existing environmental exposures can target GABBR1-centered genes. A total of 153 chemicals were extracted, among which cyclosporine, estradiol, progesterone, nicotine, and epigallocatechin gallate are drugs that have undergone clinical trials to treat diabetes and COVID-19. These clinical drug trials strongly confirm the valuable significance of GABBR1-centered network molecules in managing the coexisting diseases of diabetes and COVID-19. Thus, we have provided a novel prospect for saving the lives of people with diabetes with viral infection and valuable information to develop treatment strategies for COVID-19.

**Figure 2:**
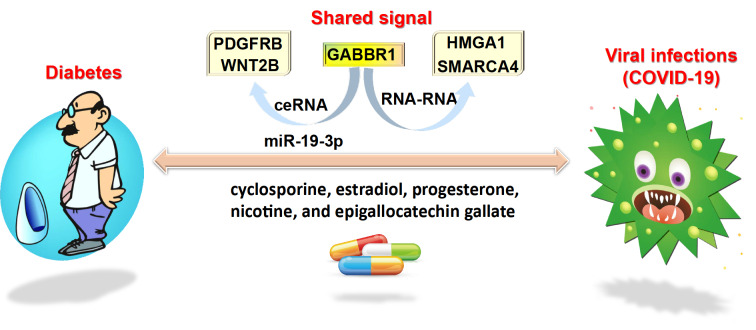
Figure 2 Visualization of the valuable information of this study The take-home message of this study is a shared GABBR1-mediated network between diabetes and viral infections that can be used to develop drugs. This network consists of ceRNA formed by PDGFRB, miR-19-3p, and WNT2B and interRNA formed by HMGA1 and SMARCA4.

## Supporting information

23375GABBR1_-_SUPPLE_DATA-5

23375GABBR1_-_SUPPLE_DATA-6

23375supplementary_method

23375Supplementary_figures

23375GABBR1_-_SUPPLE_DATA-1

23375GABBR1_-_SUPPLE_DATA-4

23375GABBR1_-_SUPPLE_DATA-2

23375GABBR1_-_SUPPLE_DATA-3

## Supplementary Data

Supplementary data is available at *Acta Biochimica et Biophysica Sinica* online.
