# Supplementary material for: Deciphering GABBR1-centered drug targets to fight viral infection with preexisting diabetes: Targeting GABBR1 for viral infection and diabetes
Source: Acta Biochim Biophys Sin (Shanghai). 2023 Nov 15;55(12):1999–2003. doi: 10.3724/abbs.2023249 (PMC10753374; doi:10.3724/abbs.2023249)
Supplement: 23375supplementary_method [file 23375supplementary_method.docx]

**Supplementary Materials and Methods**

Public resources, including online databases and tools, were recruited to collect data and perform visualization. Importantly, to ensure the objectivity of the result, all analyses throughout the study were conducted according to the default settings of the database or tool, unless otherwise specified. Each analysis method, here and the following, is explained in each database or online tool.

This study used DISEASES [1], a weekly updated resource that integrates evidence on disease-gene associations mined from literature, to query diseases related to GABBR1. ENCORI [2], an excellent online warehouse, stores more than 2.5 million miRNA-mRNA interactions from CLIP-seq and degradome-seq and RNA–RNA interactome data and 10,882 RNA-seq and 10,546 miRNA-seq data on 32 human tissues. In total, 500 ceRNA (competing endogenous RNA) and 100 interacting RNA genes of GABBR1 were downloaded from ENCORI for functional annotations. The distinguished database GEPIA [3], which provides processed gene expression and regulatory data from TCGA and GTEx databases, was used to collect 113 or 1000 coexpression genes of GABBR1 in the liver, respectively. Gene functional notes, including GAD DISEASE, GO-BP, GO-CC, GO-MF, KEGG-PATHWAY, and HIV-INTERACTION, were dissected using DAVID [4] and presented using the tool IMAGEGP. The miRNA repository TissueAtlas [5], which provides the expression information for 188 samples of 21 human organs or tissues, was used to profile the expression of the hub miRNAs in the liver. Hub miRNAs were dissected by the eyeable tool EVENN [6]. InnateDB [7], which provides registration information for 2308 innate immunity genes, was enlisted to query the hub genes. Another free-to-access repository, HPA (Human Protein Atlas) [8], which focuses on the expression data in human tissues of genes on both the mRNA and protein levels, was used to profile the single cell expression of hub genes in the liver. CTD (Comparative Toxicogenomics Database) [9], a brilliant tool designed to advance understanding about how environmental exposures affect human gene expression, was used to dissect gene–chemical interactions for the five network genes. DrugBank [10], a well-known publicly available database of clinical drugs, was used to conduct our chemical matching analysis. Gene–chemical interaction was visualized using Cytoscape [11]. The Office software EXCELL or PPT was used for general drawing.

**Acknowledgments**

We are extremely grateful to the organizations, institutions, and scientists that developed free databases and tools. Without their brilliant contributions, we could not have completed this article. As this paper involves numerous public resources, please criticize us if any incorrect references or omissions are presented here. The research scope of this paper is very wide, and a sea of literature is involved. Because of the limitation of time and space, massive superb references cannot be cited. We are just here to express our deepest wishes to these scientists.

**References**

1. Pletscher-Frankild S, Palleja A, Tsafou K, Binder JX, Jensen LJ: **DISEASES: text mining and data integration of disease-gene associations**. *Methods* 2015, **74**:83-89.

2. Li JH, Liu S, Zhou H, Qu LH, Yang JH: **starBase v2.0: decoding miRNA-ceRNA, miRNA-ncRNA and protein-RNA interaction networks from large-scale CLIP-Seq data**. *Nucleic Acids Res* 2014, **42**(Database issue):D92-97.

3. Tang Z, Kang B, Li C, Chen T, Zhang Z: **GEPIA2: an enhanced web server for large-scale expression profiling and interactive analysis**. *Nucleic Acids Res* 2019, **47**(W1):W556-W560.

4. Jiao X, Sherman BT, Huang da W, Stephens R, Baseler MW, Lane HC, Lempicki RA: **DAVID-WS: a stateful web service to facilitate gene/protein list analysis**. *Bioinformatics* 2012, **28**(13):1805-1806.

5. Ludwig N, Leidinger P, Becker K, Backes C, Fehlmann T, Pallasch C, Rheinheimer S, Meder B, Stahler C, Meese E *et al*: **Distribution of miRNA expression across human tissues**. *Nucleic Acids Res* 2016, **44**(8):3865-3877.

6. Chen T, Zhang H, Liu Y, Liu YX, Huang L: **EVenn: Easy to create repeatable and editable Venn diagrams and Venn networks online**. *J Genet Genomics* 2021, **48**(9):863-866.

7. Breuer K, Foroushani AK, Laird MR, Chen C, Sribnaia A, Lo R, Winsor GL, Hancock RE, Brinkman FS, Lynn DJ: **InnateDB: systems biology of innate immunity and beyond--recent updates and continuing curation**. *Nucleic Acids Res* 2013, **41**(Database issue):D1228-1233.

8. Uhlen M, Fagerberg L, Hallstrom BM, Lindskog C, Oksvold P, Mardinoglu A, Sivertsson A, Kampf C, Sjostedt E, Asplund A *et al*: **Proteomics. Tissue-based map of the human proteome**. *Science* 2015, **347**(6220):1260419.

9. Davis AP, Grondin CJ, Johnson RJ, Sciaky D, Wiegers J, Wiegers TC, Mattingly CJ: **Comparative Toxicogenomics Database (CTD): update 2021**. *Nucleic Acids Res* 2021, **49**(D1):D1138-D1143.

10. Wishart DS, Feunang YD, Guo AC, Lo EJ, Marcu A, Grant JR, Sajed T, Johnson D, Li C, Sayeeda Z *et al*: **DrugBank 5.0: a major update to the DrugBank database for 2018**. *Nucleic Acids Res* 2018, **46**(D1):D1074-D1082.

11. Shannon P, Markiel A, Ozier O, Baliga NS, Wang JT, Ramage D, Amin N, Schwikowski B, Ideker T: **Cytoscape: a software environment for integrated models of biomolecular interaction networks**. *Genome Res* 2003, **13**(11):2498-2504.
