# Supplementary material for: Deciphering GABBR1-centered drug targets to fight viral infection with preexisting diabetes: Targeting GABBR1 for viral infection and diabetes
Source: Acta Biochim Biophys Sin (Shanghai). 2023 Nov 15;55(12):1999–2003. doi: 10.3724/abbs.2023249 (PMC10753374; doi:10.3724/abbs.2023249)
Supplement: 23375Supplementary_figures [file 23375Supplementary_figures.pptx]

## Slide 1
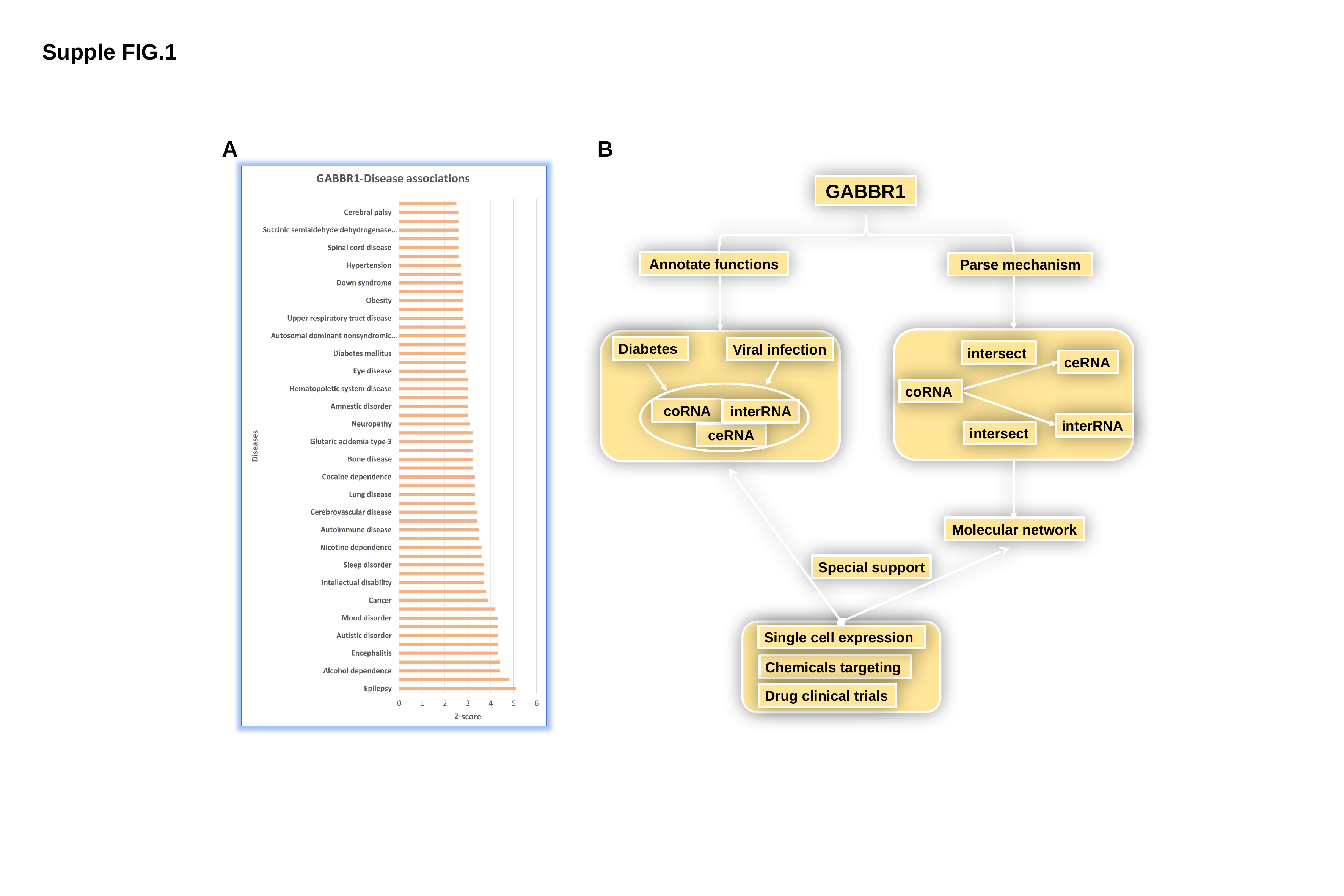

Supple FIG.1
A
B
GABBR1
Annotate functions
Parse mechanism
Diabetes
Viral infection
intersect
ceRNA
coRNA
coRNA
interRNA
interRNA
intersect
ceRNA
Molecular network
Special support
Single cell expression
Chemicals targeting
Drug clinical trials

## Slide 2
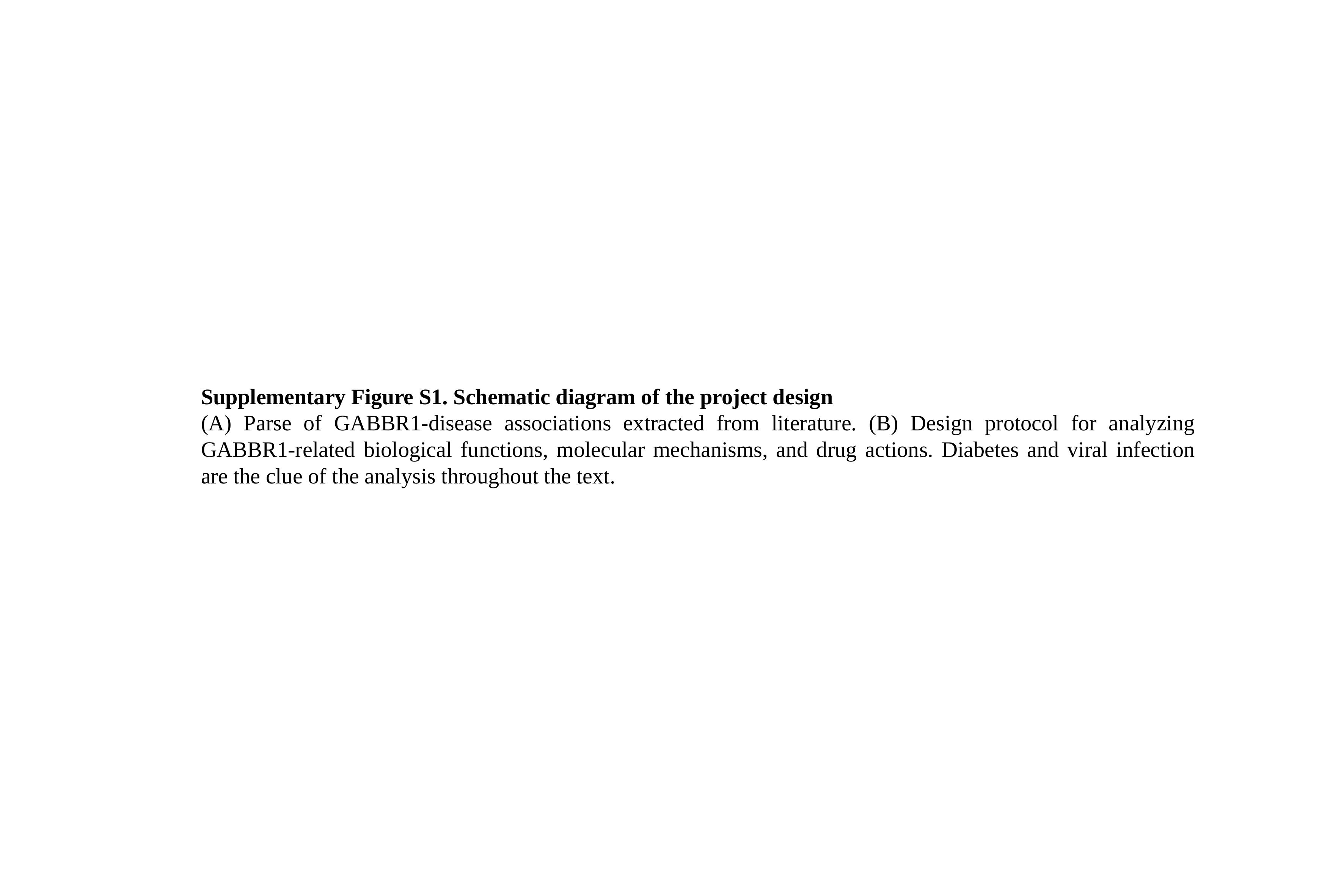

Supplementary Figure S1. Schematic diagram of the project design
(A) Parse of GABBR1-disease associations extracted from literature. (B) Design protocol for analyzing GABBR1-related biological functions, molecular mechanisms, and drug actions. Diabetes and viral infection are the clue of the analysis throughout the text.

## Slide 3
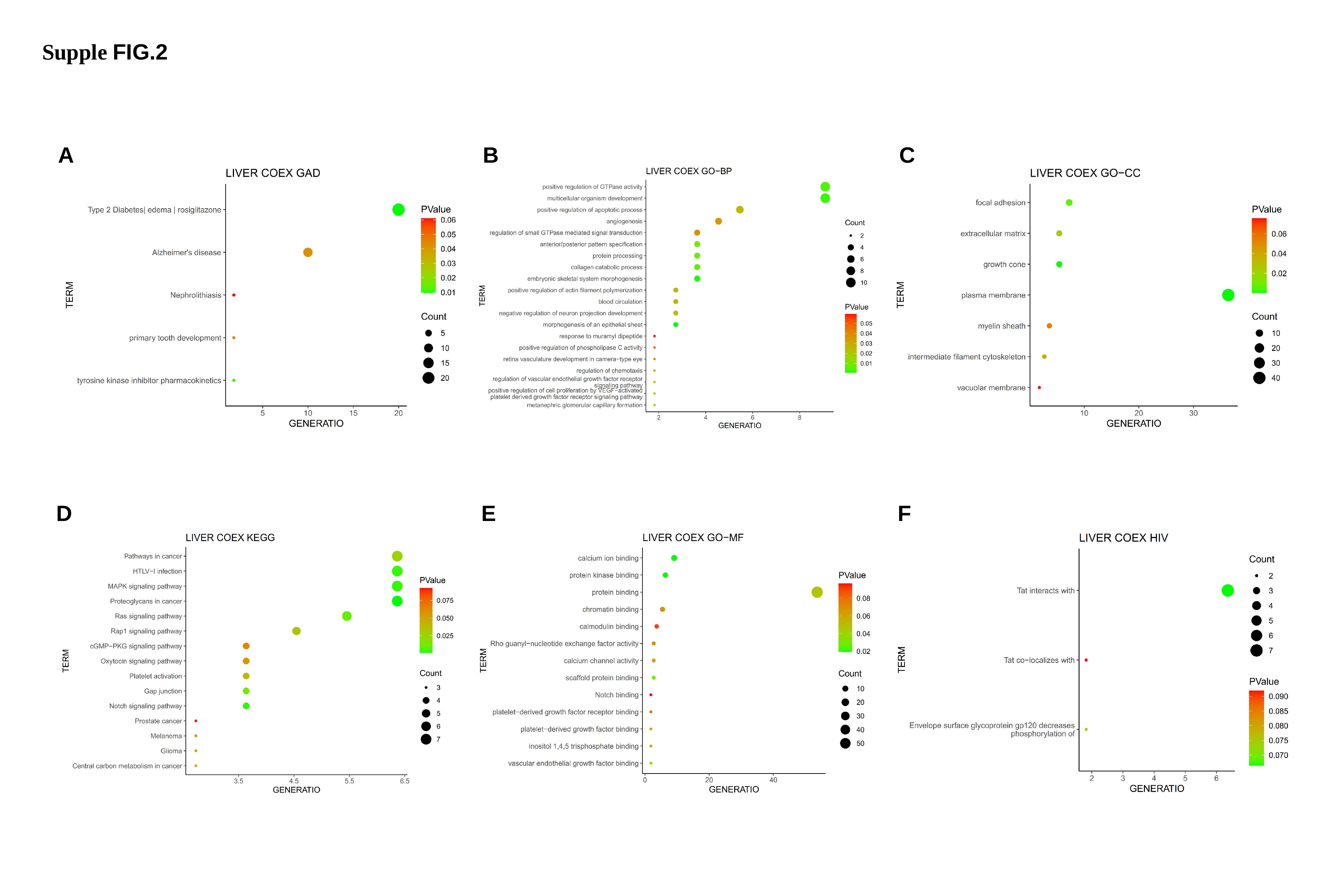

Supple FIG.2
A
B
C
D
E
F

## Slide 4
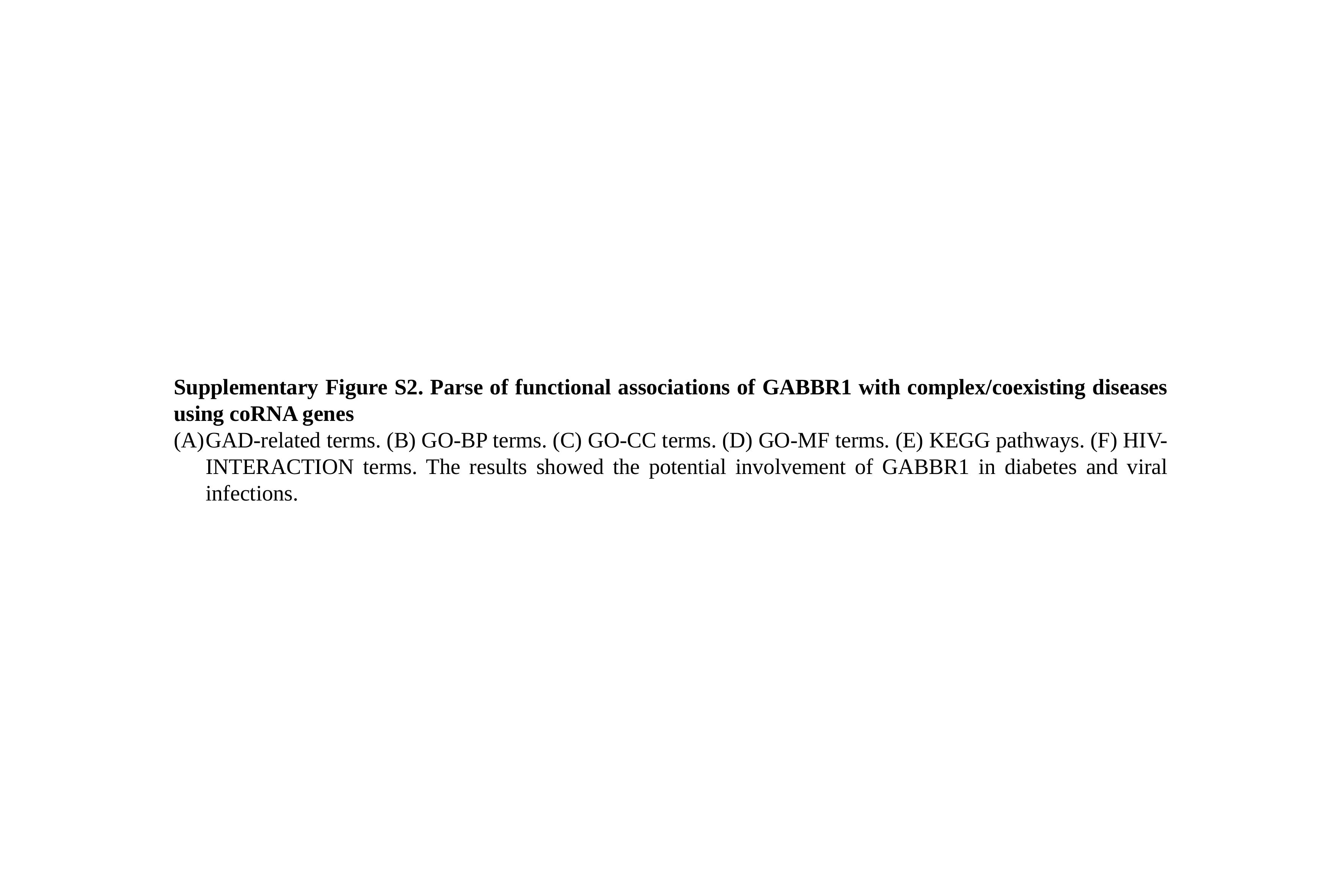

Supplementary Figure S2. Parse of functional associations of GABBR1 with complex/coexisting diseases using coRNA genes
GAD-related terms. (B) GO-BP terms. (C) GO-CC terms. (D) GO-MF terms. (E) KEGG pathways. (F) HIV-INTERACTION terms. The results showed the potential involvement of GABBR1 in diabetes and viral infections.

## Slide 5
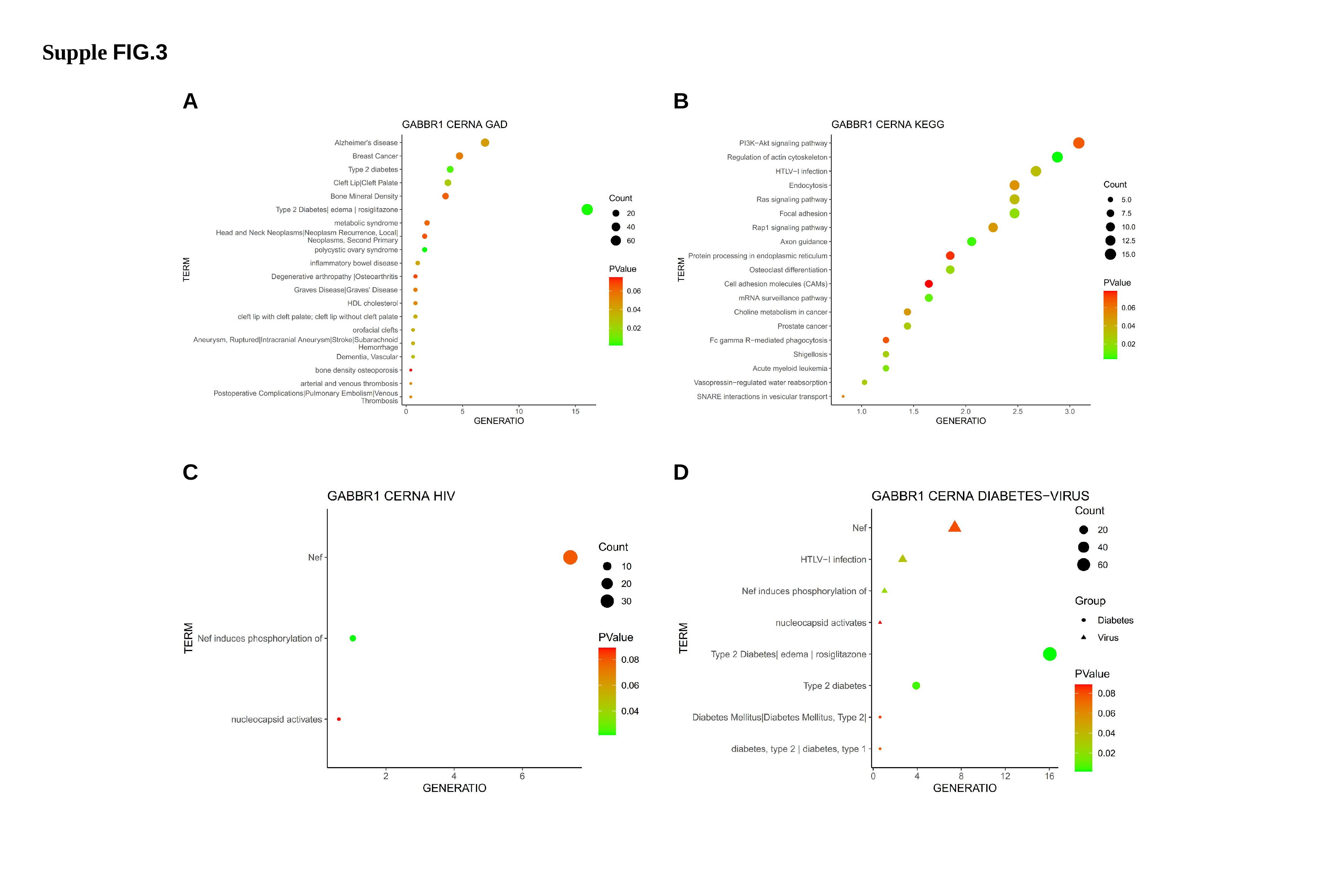

Supple FIG.3
A
B
C
D

## Slide 6
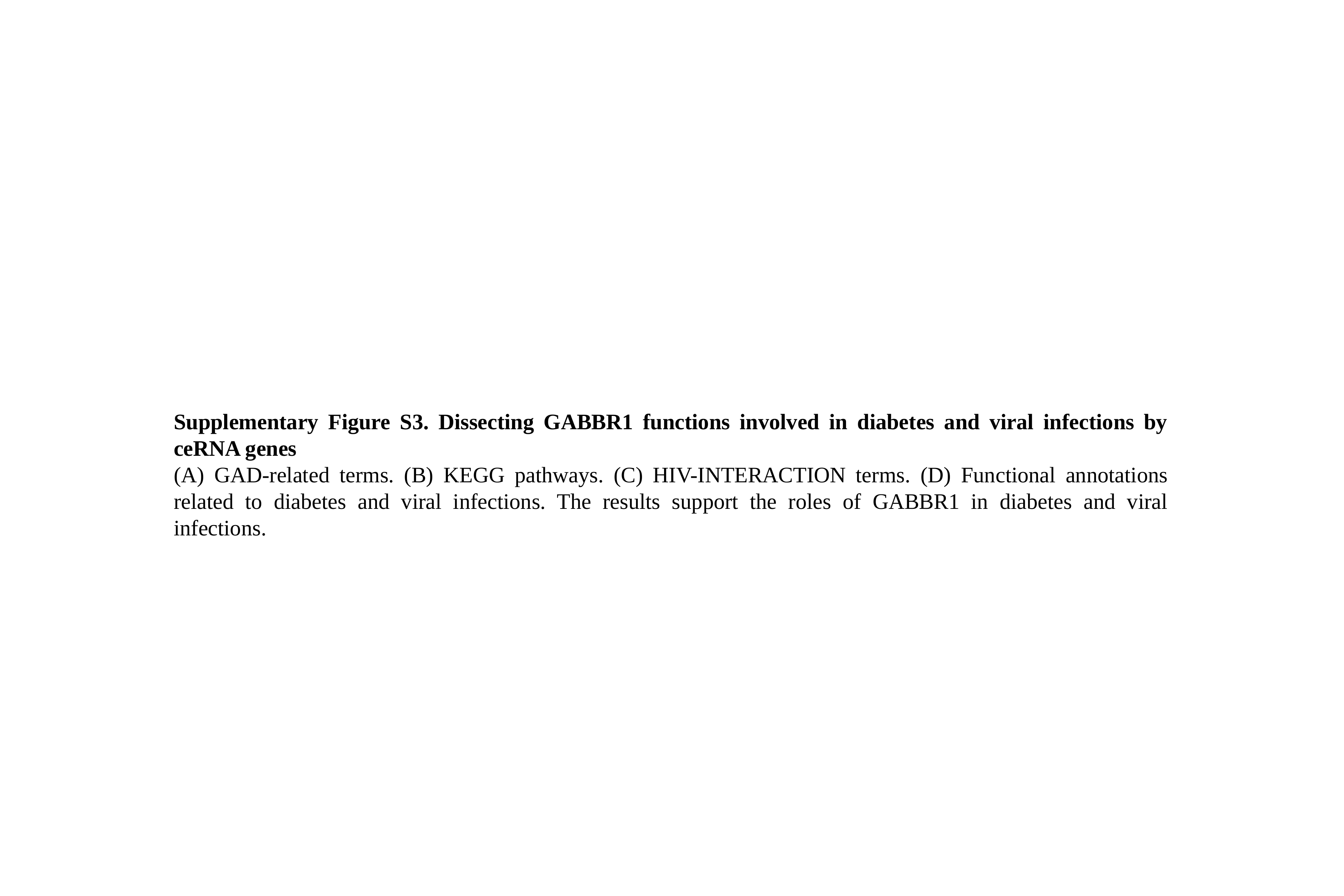

Supplementary Figure S3. Dissecting GABBR1 functions involved in diabetes and viral infections by ceRNA genes
(A) GAD-related terms. (B) KEGG pathways. (C) HIV-INTERACTION terms. (D) Functional annotations related to diabetes and viral infections. The results support the roles of GABBR1 in diabetes and viral infections.

## Slide 7
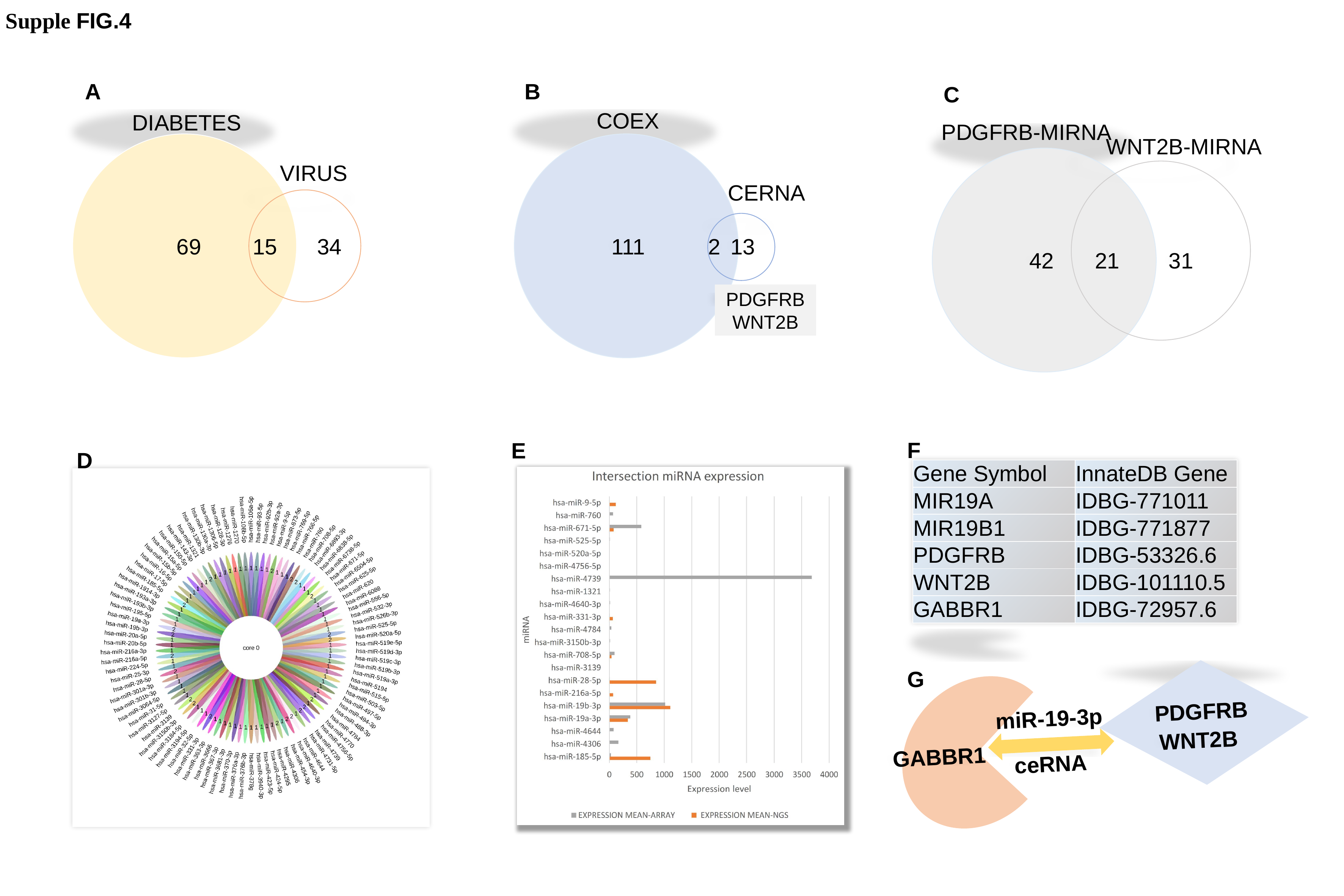

Supple FIG.4
A
B
C
COEX
DIABETES
PDGFRB-MIRNA
WNT2B-MIRNA
VIRUS
CERNA
69
15
34
111
2
13
42
21
31
PDGFRB
WNT2B
F
E
D
| Gene Symbol | InnateDB Gene |
| --- | --- |
| MIR19A | IDBG-771011 |
| MIR19B1 | IDBG-771877 |
| PDGFRB | IDBG-53326.6 |
| WNT2B | IDBG-101110.5 |
| GABBR1 | IDBG-72957.6 |
PDGFRB
miR-19-3p
WNT2B
GABBR1
ceRNA
G

## Slide 8
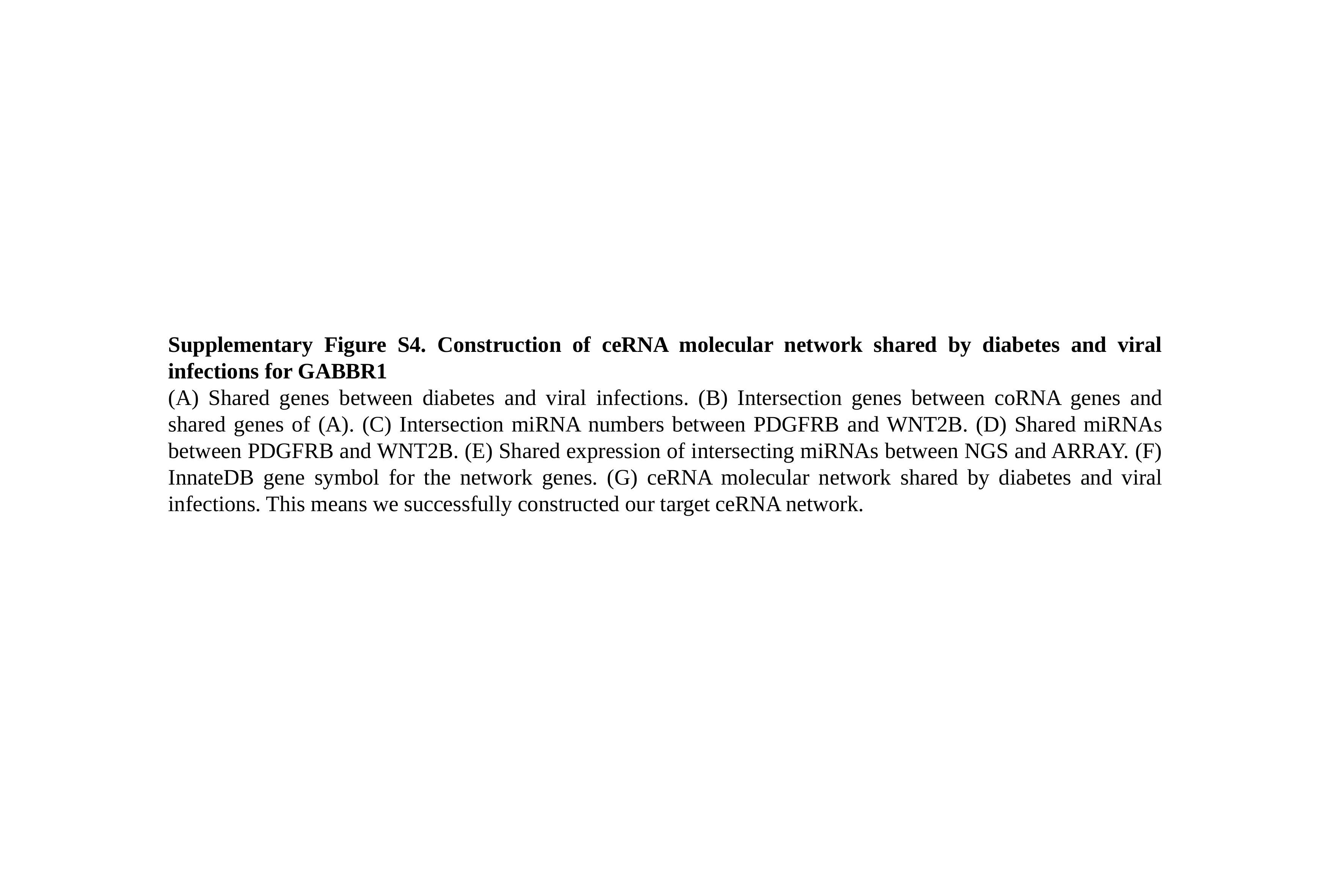

Supplementary Figure S4. Construction of ceRNA molecular network shared by diabetes and viral infections for GABBR1
(A) Shared genes between diabetes and viral infections. (B) Intersection genes between coRNA genes and shared genes of (A). (C) Intersection miRNA numbers between PDGFRB and WNT2B. (D) Shared miRNAs between PDGFRB and WNT2B. (E) Shared expression of intersecting miRNAs between NGS and ARRAY. (F) InnateDB gene symbol for the network genes. (G) ceRNA molecular network shared by diabetes and viral infections. This means we successfully constructed our target ceRNA network.

## Slide 9
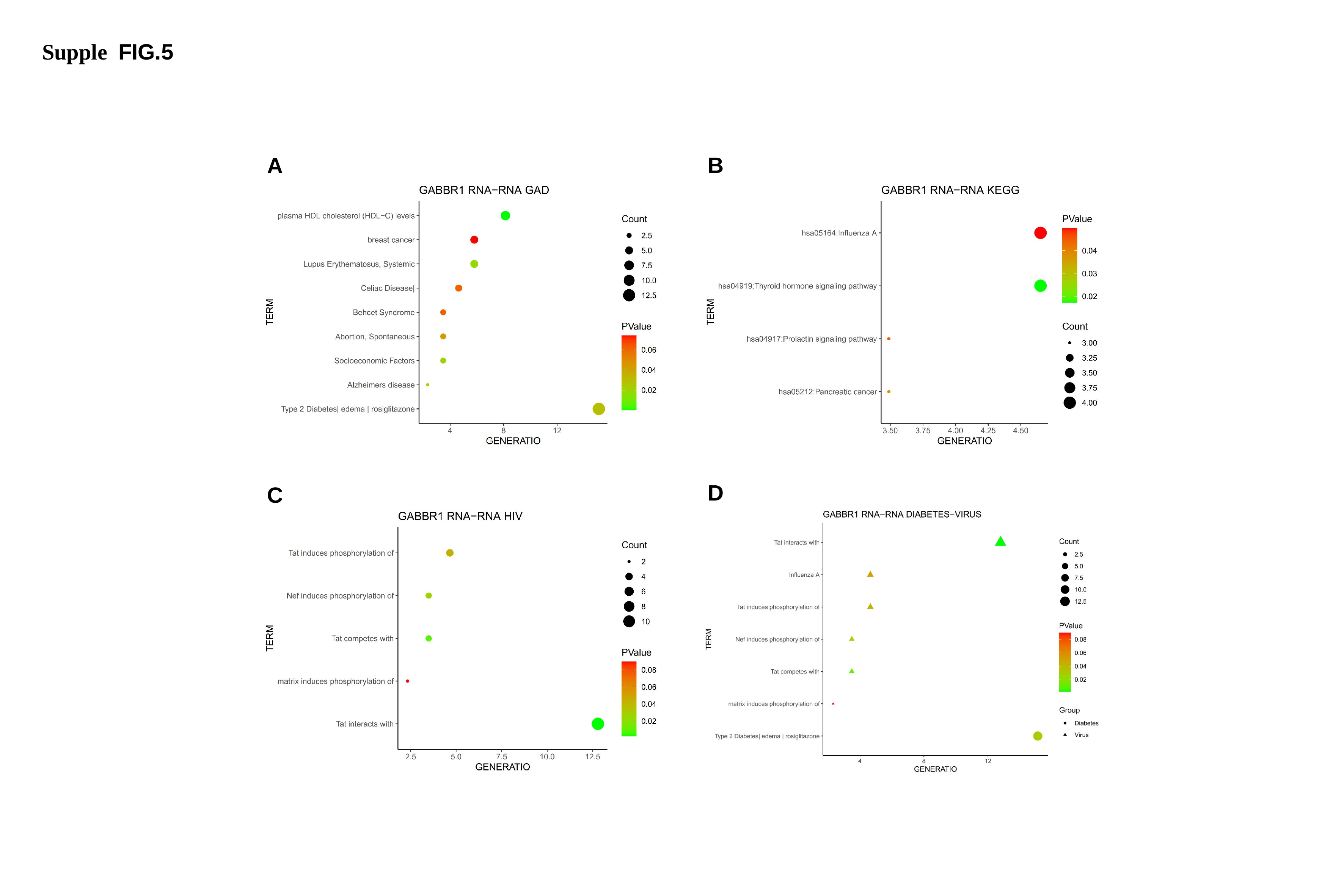

Supple FIG.5
B
A
D
C

## Slide 10
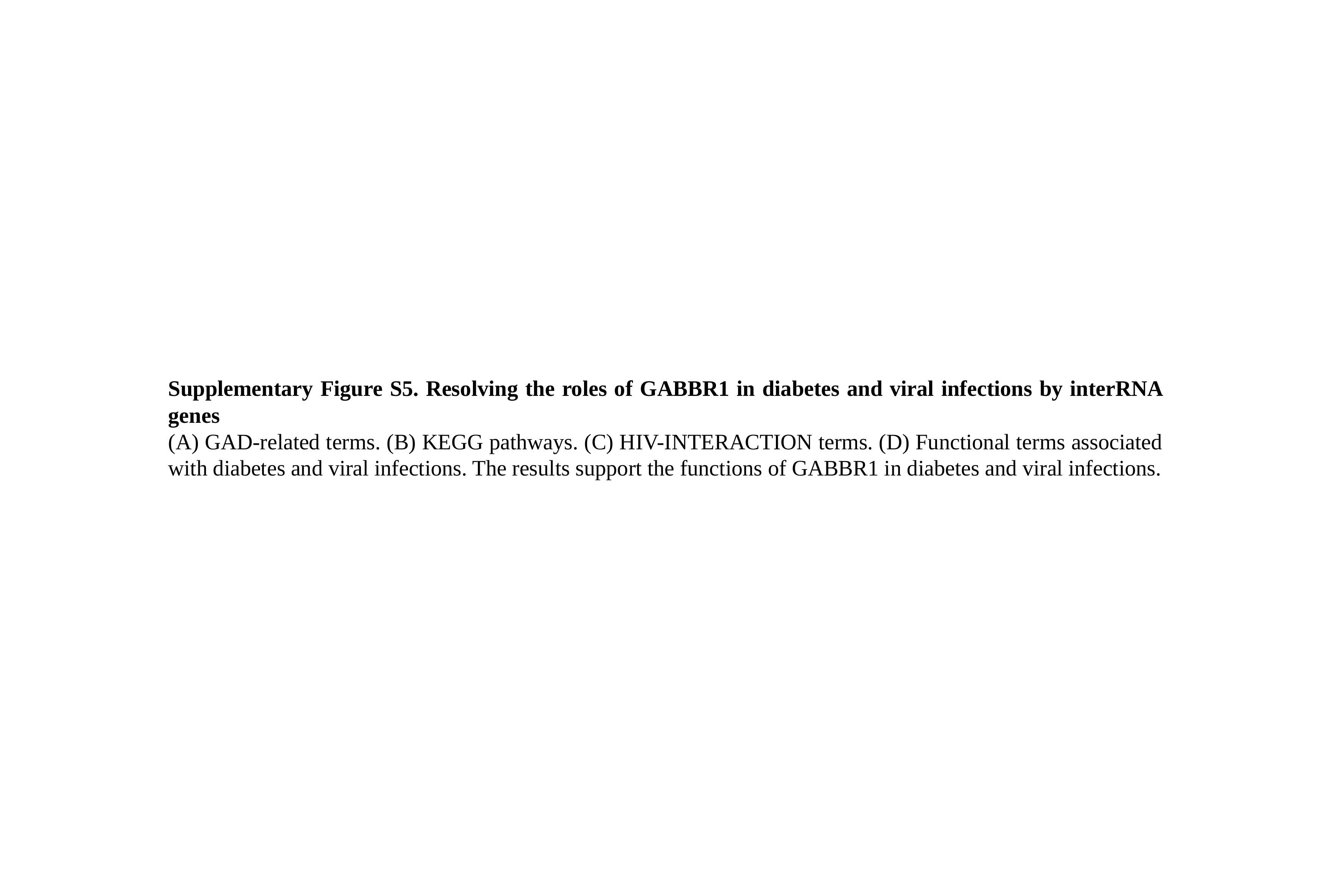

Supplementary Figure S5. Resolving the roles of GABBR1 in diabetes and viral infections by interRNA genes
(A) GAD-related terms. (B) KEGG pathways. (C) HIV-INTERACTION terms. (D) Functional terms associated with diabetes and viral infections. The results support the functions of GABBR1 in diabetes and viral infections.

## Slide 11
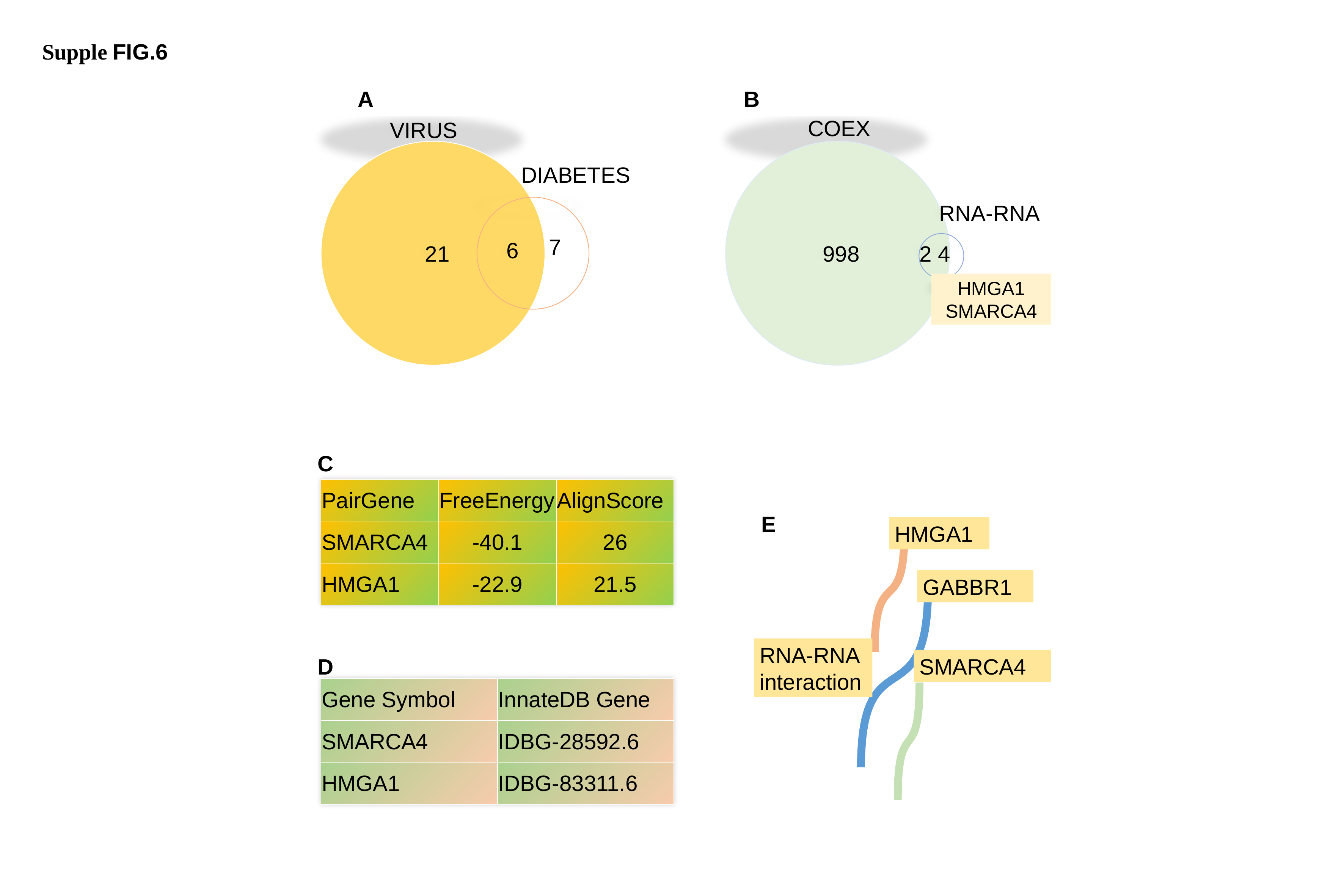

Supple FIG.6
A
B
COEX
VIRUS
DIABETES
RNA-RNA
7
6
21
998
2
4
HMGA1
SMARCA4
C
| PairGene | FreeEnergy | AlignScore |
| --- | --- | --- |
| SMARCA4 | -40.1 | 26 |
| HMGA1 | -22.9 | 21.5 |
E
HMGA1
GABBR1
RNA-RNA interaction
SMARCA4
D
| Gene Symbol | InnateDB Gene |
| --- | --- |
| SMARCA4 | IDBG-28592.6 |
| HMGA1 | IDBG-83311.6 |

## Slide 12
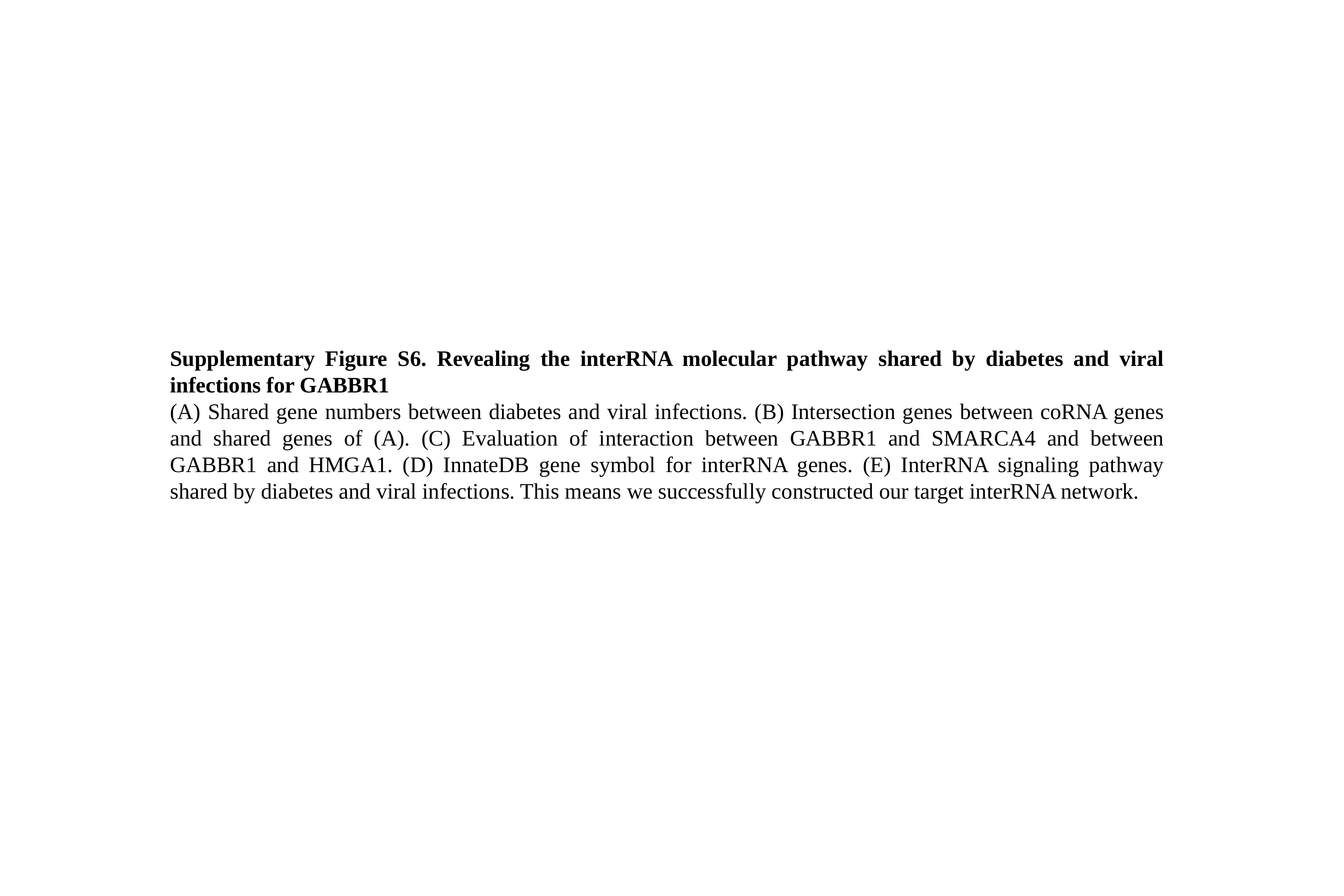

Supplementary Figure S6. Revealing the interRNA molecular pathway shared by diabetes and viral infections for GABBR1
(A) Shared gene numbers between diabetes and viral infections. (B) Intersection genes between coRNA genes and shared genes of (A). (C) Evaluation of interaction between GABBR1 and SMARCA4 and between GABBR1 and HMGA1. (D) InnateDB gene symbol for interRNA genes. (E) InterRNA signaling pathway shared by diabetes and viral infections. This means we successfully constructed our target interRNA network.

## Slide 13
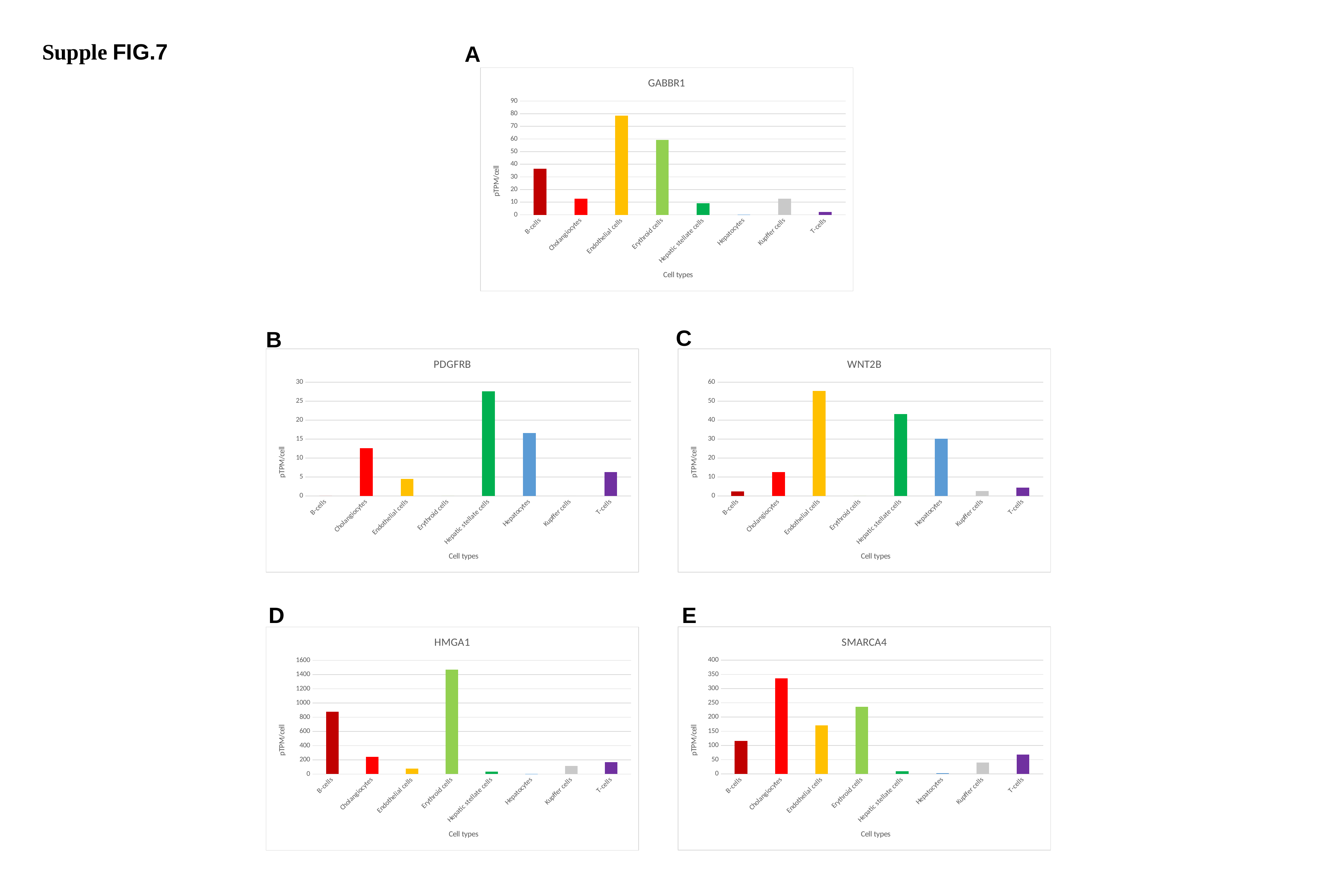

Supple FIG.7
A
### Chart:
| Category | GABBR1 |
|---|---|
| B-cells | 36.600306278713624 |
| Cholangiocytes | 12.605042016806724 |
| Endothelial cells | 78.39195979899498 |
| Erythroid cells | 59.34065934065934 |
| Hepatic stellate cells | 9.22844175491679 |
| Hepatocytes | 0.28506271379700004 |
| Kupffer cells | 12.61564339781329 |
| T-cells | 2.228826151560179 |C
B
### Chart:
| Category | PDGFRB |
|---|---|
| B-cells | 0.0 |
| Cholangiocytes | 12.605042016806724 |
| Endothelial cells | 4.522613065326633 |
| Erythroid cells | 0.0 |
| Hepatic stellate cells | 27.534039334341898 |
| Hepatocytes | 16.590649942987447 |
| Kupffer cells | 0.0 |
| T-cells | 6.2902426944031715 |
### Chart:
| Category | WNT2B |
|---|---|
| B-cells | 2.4502297090352214 |
| Cholangiocytes | 12.605042016806724 |
| Endothelial cells | 55.27638190954775 |
| Erythroid cells | 0.0 |
| Hepatic stellate cells | 43.116490166414515 |
| Hepatocytes | 30.24515393386545 |
| Kupffer cells | 2.5231286795626584 |
| T-cells | 4.408122833085685 |D
E
### Chart:
| Category | SMARCA4 |
|---|---|
| B-cells | 116.07963246554367 |
| Cholangiocytes | 336.1344537815126 |
| Endothelial cells | 170.35175879396985 |
| Erythroid cells | 236.26373626373623 |
| Hepatic stellate cells | 9.682299546142211 |
| Hepatocytes | 2.4230330672748006 |
| Kupffer cells | 39.78132884777124 |
| T-cells | 68.69737493808815 |
### Chart:
| Category | HMGA1 |
|---|---|
| B-cells | 877.947932618683 |
| Cholangiocytes | 240.33613445378154 |
| Endothelial cells | 78.39195979899498 |
| Erythroid cells | 1471.428571428572 |
| Hepatic stellate cells | 34.03933434190621 |
| Hepatocytes | 0.7126567844925885 |
| Kupffer cells | 116.23212783851974 |
| T-cells | 166.07231302625058 |

## Slide 14
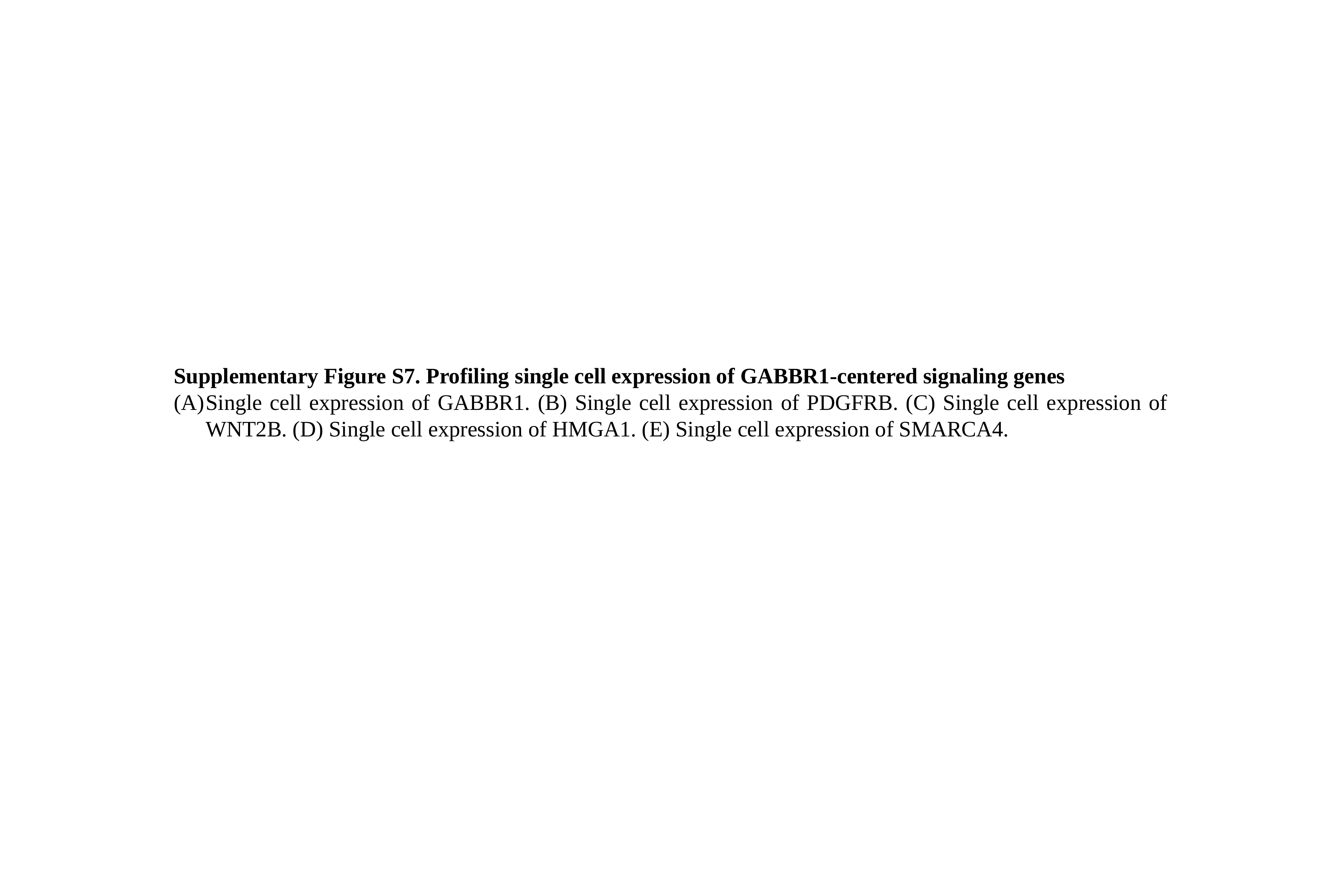

Supplementary Figure S7. Profiling single cell expression of GABBR1-centered signaling genes
Single cell expression of GABBR1. (B) Single cell expression of PDGFRB. (C) Single cell expression of WNT2B. (D) Single cell expression of HMGA1. (E) Single cell expression of SMARCA4.
